# (2*S*,N*S*)-*N*-Allyl-*N*-benzyl-1-hydr­oxy-3-(4-hydroxy­phen­yl)-*N*-methyl­propan-2-aminium bromide

**DOI:** 10.1107/S1600536808023210

**Published:** 2008-07-31

**Authors:** Hua-Fang Wu, Ying-Gang Luo, Kai-Bei Yu, Guo-Lin Zhang, Xin-Fu Pan

**Affiliations:** a Department of Chemistry, State Key Laboratory of Applied Organic Chemistry, Lanzhou University, Lanzhou 730000, People’s Republic of China; b Chengdu Institute of Biology, Chinese Academy of Sciences, Chengdu 610041, People’s Republic of China; c Chengdu Institute of Organic Chemistry, Chinese Academy of Sciences, Chengdu 610041, People’s Republic of China

## Abstract

The title compound, C_20_H_26_NO_2_^+^·Br^−^, is an *N*-chiral quaternary ammonium salt synthesized from (2S*)-*N*-benzyl-*N*-methyl­tyrosine methyl ester. The dihedral angle between the phenyl ring and the benzene ring is 11.61 (19)°. In the crystal structure, the allyl group is disordered over two positions with site occupancy factors of *ca* 0.8 and 0.2. The bromide anion links to the quaternary ammonium cations *via* O—H⋯Br hydrogen bonding. An intramolecular O—H⋯Br hydrogen bond is also observed.

## Related literature

For general background, see: Maruoka & Ooi (2003 ▶); Ooi & Maruoka (2007 ▶). For a related structure, see: Tayama & Tanaka (2007 ▶). For synthesis, see: White & Konopelski (2005 ▶).
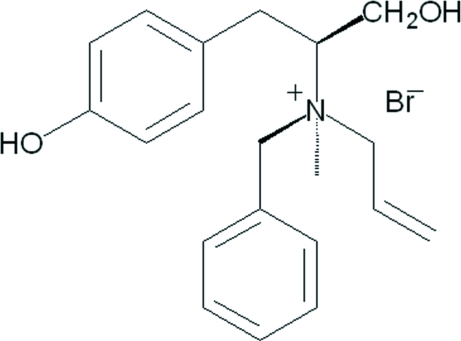


## Experimental

### 

#### Crystal data

C_20_H_26_NO_2_^+^·Br^−^
*M* *_r_* = 392.33
Orthorhombic, 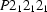

*a* = 10.3716 (10) Å
*b* = 12.1566 (10) Å
*c* = 15.6790 (16) Å
*V* = 1976.9 (3) Å^3^
*Z* = 4
Mo *K*α radiation
μ = 2.09 mm^−1^
*T* = 293 (2) K
0.45 × 0.43 × 0.40 mm

#### Data collection

Rigaku R-AXIS RAPID IP diffractometer
Absorption correction: multi-scan (*ABSCOR*; Higashi, 1995 ▶) *T*_min_ = 0.406, *T*_max_ = 0.433
18872 measured reflections
4524 independent reflections
2606 reflections with *I* > 2σ(*I*)
*R*_int_ = 0.057

#### Refinement

*R*[*F* ^2^ > 2σ(*F* ^2^)] = 0.054
*wR*(*F* ^2^) = 0.211
*S* = 1.00
4524 reflections
225 parameters
2 restraints
H-atom parameters constrained
Δρ_max_ = 0.89 e Å^−3^
Δρ_min_ = −0.69 e Å^−3^
Absolute structure: Flack (1983 ▶), with 1949 Friedel pairs
Flack parameter: 0.009 (19)

### 

Data collection: *RAPID-AUTO* (Rigaku, 2004 ▶); cell refinement: *RAPID-AUTO*; data reduction: *RAPID-AUTO*; program(s) used to solve structure: *SHELXTL* (Sheldrick, 2008 ▶); program(s) used to refine structure: *SHELXTL*; molecular graphics: *SHELXTL*; software used to prepare material for publication: *SHELXTL*.

## Supplementary Material

Crystal structure: contains datablocks global, I. DOI: 10.1107/S1600536808023210/xu2435sup1.cif

Structure factors: contains datablocks I. DOI: 10.1107/S1600536808023210/xu2435Isup2.hkl

Additional supplementary materials:  crystallographic information; 3D view; checkCIF report

## Figures and Tables

**Table 1:** Hydrogen-bond geometry (Å, °)

*D*—H⋯*A*	*D*—H	H⋯*A*	*D*⋯*A*	*D*—H⋯*A*
O1—H1O⋯Br1	0.82	2.43	3.231 (4)	167
O2—H2O⋯Br1^i^	0.82	2.38	3.192 (5)	171

Symmetry code: (i) 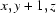.

**Fig. 1.**
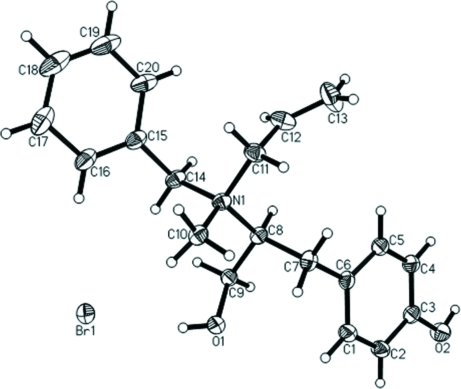
The molecular structure of the title compound, displacement ellipsoids are drawn at the 30% probability level. The minor disordered component has been omitted for clarity.

**Fig. 2.**
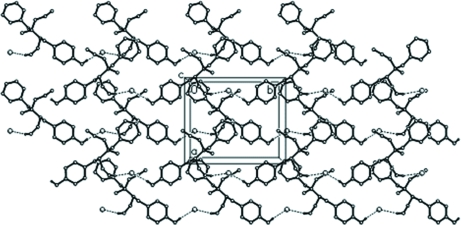
The unit cell packing diagram of the title compound. Dashed lines indicate hydrogen bonding.

## supplementary crystallographic information

### Comment

As an important class of asymmetric catalysts of phase-transfer catalysts, chiral quaternary ammonium salts show great application in asymmetric organic synthesis (Maruoka & Ooi, 2003; Ooi & Maruoka, 2007). The title compound is a N-chiral quaternary ammonium salt (N-CQAS), we present here its structure.

The molecular structure is shown in Fig. 1. The quaternary ammonium cation displays an extended structure, the C14—N1—C8—C7 torsion angle is 179.3 (5)°. The terminal C1-benzene and C15-phenyl rings are nearly parallel to each other [dihedral angle 11.61 (19)°], and approximately perpendicular to the central C7/C6/N1/C14 mean plane with dihedral angles of 85.1 (3) and 88.8 (4)°, respectively. Bond lengths and angles agree with those found in a reported N-chiral quaternary ammonium salt (Tayama & Tanaka, 2007).

In the crystal structure the Br^-^ anion links with the quaternary ammonium cations *via* O—H···Br hydrogen bonding (Table 1), to form the one dimensional supra-molecular structure along the *b* axis (Fig. 2).

### Experimental

(2*S**)-*N*-Benzyl-*N*-methyltyrosine methyl ester (White & Konopelski, 2005) (1 mmol) was reduced by lithium aluminium hydride (1 mmol) to afford (2*S**)-*N*-Benzyl-*N*-methyl-2-amino-3-(4-hydroxyphenyl)propan-1-ol, which was then dissolved in absolute acetonitrile (5 ml), and allyl bromide (2 mmol) was added. The mixture was heated to reflux for 42 h. After being cooled to room temperature, the excess allyl bromide and acetonitrile were removed under reduced pressure. The residue was purified by flash chromatography eluted with ethyl acetate/methanol (8:1) to afford the diastereomeric mixture of the N-CQAS with yields (90%). The mixture was recrystallized from ethanol to afford the single crystals of the title compound. Yield (41%).

### Refinement

H atoms were placed in calculated positions with C—H = 0.93–0.98 Å, O—H = 0.82 Å, and refined in riding mode with *U*_iso_(H) = 1.2*U*_eq_(C,O). The terminal carbon (C13) atom of the allyl group is disordered over two sites, occupancies were refined and converged to 0.778:0.222.

### Crystal data

**Table:**

C_20_H_26_NO_2_^+^·Br^–^	*D*_x_ = 1.318 Mg m^−^^3^
*M**_r_* = 392.33	Melting point: 449(5) K
Orthorhombic, *P*2_1_2_1_2_1_	Mo *K*α radiation λ = 0.71073 Å
Hall symbol: P 2ac 2ab	Cell parameters from 10762 reflections
*a* = 10.3716 (10) Å	θ = 3.1–27.4º
*b* = 12.1566 (10) Å	µ = 2.09 mm^−^^1^
*c* = 15.6790 (16) Å	*T* = 293 (2) K
*V* = 1976.9 (3) Å^3^	Block, colourless
*Z* = 4	0.45 × 0.43 × 0.40 mm
*F*_000_ = 816	

### Data collection

**Table:**

Rigaku R-AXIS RAPID IP diffractometer	4524 independent reflections
Radiation source: Rotating anode	2606 reflections with *I* > 2σ(*I*)
Monochromator: graphite	*R*_int_ = 0.057
*T* = 293(2) K	θ_max_ = 27.5º
ω scans	θ_min_ = 3.1º
Absorption correction: multi-scan(ABSCOR; Higashi, 1995)	*h* = −13→13
*T*_min_ = 0.406, *T*_max_ = 0.434	*k* = −15→15
18872 measured reflections	*l* = −20→20

### Refinement

**Table:**

Refinement on *F*^2^	Hydrogen site location: inferred from neighbouring sites
Least-squares matrix: full	H-atom parameters constrained
*R*[*F*^2^ > 2σ(*F*^2^)] = 0.054	*w* = 1/[σ^2^(*F*_o_^2^) + (0.128*P*)^2^ + 0.338*P*] where *P* = (*F*_o_^2^ + 2*F*_c_^2^)/3
*wR*(*F*^2^) = 0.211	(Δ/σ)_max_ = 0.001
*S* = 1.00	Δρ_max_ = 0.89 e Å^−^^3^
4524 reflections	Δρ_min_ = −0.69 e Å^−^^3^
225 parameters	Extinction correction: SHELXTL (Sheldrick, 2008), Fc^*^=kFc[1+0.001xFc^2^λ^3^/sin(2θ)]^-1/4^
2 restraints	Extinction coefficient: 0.058 (6)
Primary atom site location: structure-invariant direct methods	Absolute structure: Flack (1983), with 1949 Friedel pairs
Secondary atom site location: difference Fourier map	Flack parameter: 0.009 (19)

### Special details

**Table:**

Experimental. IR (KBr): 3297, 3034, 1612, 1515, 1464, 1264, 1058, 850 (cm^-1^); ^13^CNMR (150 MHz, DMSO-d_6_, δ, p.p.m.): 156.8, 133.7, 130.8, 129.4, 128.5, 127.4, 126.9, 126.8, 116.0, 73.9, 63.7, 62.9, 56.6, 47.0, 29.6.
Geometry. All e.s.d.'s (except the e.s.d. in the dihedral angle between two l.s. planes) are estimated using the full covariance matrix. The cell e.s.d.'s are taken into account individually in the estimation of e.s.d.'s in distances, angles and torsion angles; correlations between e.s.d.'s in cell parameters are only used when they are defined by crystal symmetry. An approximate (isotropic) treatment of cell e.s.d.'s is used for estimating e.s.d.'s involving l.s. planes.
Refinement. Refinement of *F*^2^ against ALL reflections. The weighted *R*-factor *wR* and goodness of fit *S* are based on *F*^2^, conventional *R*-factors *R* are based on *F*, with *F* set to zero for negative *F*^2^. The threshold expression of *F*^2^ > σ(*F*^2^) is used only for calculating *R*-factors(gt) *etc*. and is not relevant to the choice of reflections for refinement. *R*-factors based on *F*^2^ are statistically about twice as large as those based on *F*, and *R*- factors based on ALL data will be even larger.

### Fractional atomic coordinates and isotropic or equivalent isotropic displacement parameters (Å^2^)

**Table:**

	*x*	*y*	*z*	*U*_iso_*/*U*_eq_	Occ. (<1)
Br1	0.63828 (7)	0.06624 (5)	0.31732 (5)	0.0766 (3)	
O1	0.6728 (4)	0.3120 (4)	0.3923 (3)	0.0748 (12)	
H1O	0.6764	0.2479	0.3762	0.090*	
O2	0.7918 (4)	0.8588 (4)	0.3897 (4)	0.0863 (14)	
H2O	0.7452	0.9091	0.3736	0.104*	
N1	0.3651 (4)	0.3213 (4)	0.4017 (3)	0.0552 (10)	
C1	0.7120 (6)	0.5784 (6)	0.4504 (5)	0.0739 (17)	
H1	0.7557	0.5151	0.4668	0.089*	
C2	0.7823 (6)	0.6731 (5)	0.4309 (5)	0.0739 (18)	
H2	0.8718	0.6726	0.4334	0.089*	
C3	0.7171 (6)	0.7673 (5)	0.4080 (4)	0.0680 (16)	
C4	0.5853 (6)	0.7668 (5)	0.4011 (4)	0.0696 (16)	
H4	0.5420	0.8296	0.3830	0.083*	
C5	0.5173 (6)	0.6733 (5)	0.4210 (5)	0.0667 (16)	
H5	0.4278	0.6747	0.4178	0.080*	
C6	0.5784 (5)	0.5767 (5)	0.4459 (4)	0.0595 (13)	
C7	0.5053 (7)	0.4747 (5)	0.4658 (4)	0.0666 (15)	
H7A	0.4282	0.4936	0.4976	0.080*	
H7B	0.5578	0.4271	0.5013	0.080*	
C8	0.4670 (5)	0.4131 (4)	0.3849 (4)	0.0538 (12)	
H8	0.4261	0.4670	0.3471	0.065*	
C9	0.5867 (5)	0.3719 (5)	0.3389 (4)	0.0576 (13)	
H9A	0.5603	0.3251	0.2919	0.069*	
H9B	0.6322	0.4345	0.3149	0.069*	
C10	0.4099 (6)	0.2377 (5)	0.4638 (4)	0.0606 (13)	
H10A	0.3393	0.1905	0.4790	0.073*	
H10B	0.4419	0.2738	0.5140	0.073*	
H10C	0.4775	0.1947	0.4386	0.073*	
C11	0.2426 (6)	0.3716 (6)	0.4391 (4)	0.0707 (16)	
H11A	0.1774	0.3147	0.4435	0.085*	
H11B	0.2611	0.3974	0.4964	0.085*	
C12	0.1902 (7)	0.4635 (6)	0.3892 (5)	0.092 (2)	0.778 (18)
H12	0.1973	0.4594	0.3302	0.111*	0.778 (18)
C13	0.1339 (16)	0.5511 (10)	0.4216 (8)	0.132 (6)	0.778 (18)
H13A	0.1250	0.5581	0.4804	0.158*	0.778 (18)
H13B	0.1030	0.6060	0.3857	0.158*	0.778 (18)
C12'	0.1902 (7)	0.4635 (6)	0.3892 (5)	0.092 (2)	0.222 (18)
H12'	0.2394	0.5064	0.3525	0.111*	0.222 (18)
C13'	0.0657 (12)	0.479 (5)	0.402 (3)	0.132 (6)	0.222 (18)
H13C	0.0212	0.4331	0.4396	0.158*	0.222 (18)
H13D	0.0225	0.5346	0.3736	0.158*	0.222 (18)
C14	0.3330 (5)	0.2665 (5)	0.3165 (4)	0.0644 (14)	
H14A	0.3090	0.3235	0.2762	0.077*	
H14B	0.4106	0.2317	0.2950	0.077*	
C15	0.2279 (6)	0.1823 (6)	0.3179 (5)	0.0728 (17)	
C16	0.2603 (7)	0.0727 (7)	0.3287 (5)	0.086 (2)	
H16	0.3459	0.0529	0.3372	0.103*	
C17	0.1623 (10)	−0.0099 (9)	0.3266 (6)	0.111 (3)	
H17	0.1820	−0.0839	0.3346	0.133*	
C18	0.0386 (10)	0.0242 (12)	0.3125 (7)	0.121 (4)	
H18	−0.0263	−0.0285	0.3114	0.145*	
C19	0.0055 (9)	0.1317 (14)	0.3001 (7)	0.119 (4)	
H19	−0.0799	0.1514	0.2902	0.143*	
C20	0.1000 (6)	0.2092 (8)	0.3026 (5)	0.093 (2)	
H20	0.0781	0.2826	0.2938	0.112*	

### Atomic displacement parameters (Å^2^)

**Table:**

	*U*^11^	*U*^22^	*U*^33^	*U*^12^	*U*^13^	*U*^23^
Br1	0.0760 (4)	0.0657 (4)	0.0881 (5)	0.0026 (3)	−0.0014 (4)	−0.0123 (3)
O1	0.066 (2)	0.060 (2)	0.098 (3)	0.004 (2)	−0.012 (2)	−0.007 (2)
O2	0.069 (3)	0.062 (2)	0.127 (4)	−0.015 (2)	0.000 (3)	0.015 (3)
N1	0.048 (2)	0.066 (3)	0.051 (2)	−0.006 (2)	0.003 (2)	0.0028 (19)
C1	0.068 (4)	0.060 (4)	0.093 (5)	0.006 (3)	−0.008 (3)	0.002 (4)
C2	0.061 (3)	0.055 (3)	0.105 (5)	0.001 (3)	−0.007 (3)	0.008 (3)
C3	0.066 (3)	0.057 (3)	0.081 (4)	−0.014 (3)	0.001 (3)	−0.001 (3)
C4	0.068 (3)	0.057 (3)	0.083 (4)	−0.001 (3)	−0.008 (3)	0.000 (3)
C5	0.061 (3)	0.059 (3)	0.081 (4)	−0.007 (3)	0.002 (3)	−0.004 (3)
C6	0.065 (3)	0.048 (3)	0.065 (3)	−0.005 (3)	0.002 (3)	−0.004 (3)
C7	0.076 (4)	0.059 (3)	0.065 (4)	−0.007 (3)	0.002 (3)	−0.002 (3)
C8	0.055 (3)	0.053 (3)	0.054 (3)	−0.009 (2)	0.001 (2)	0.007 (2)
C9	0.055 (3)	0.054 (3)	0.064 (3)	−0.002 (2)	0.003 (2)	0.001 (2)
C10	0.067 (3)	0.055 (3)	0.060 (3)	−0.012 (3)	−0.001 (3)	0.012 (2)
C11	0.060 (3)	0.087 (4)	0.065 (4)	0.011 (3)	0.009 (3)	−0.003 (3)
C12	0.063 (4)	0.115 (7)	0.099 (5)	0.019 (4)	0.010 (4)	0.011 (5)
C13	0.178 (14)	0.128 (11)	0.089 (7)	0.077 (12)	−0.012 (8)	−0.028 (7)
C12'	0.063 (4)	0.115 (7)	0.099 (5)	0.019 (4)	0.010 (4)	0.011 (5)
C13'	0.178 (14)	0.128 (11)	0.089 (7)	0.077 (12)	−0.012 (8)	−0.028 (7)
C14	0.057 (3)	0.086 (4)	0.050 (3)	−0.013 (3)	0.000 (3)	−0.004 (3)
C15	0.059 (3)	0.097 (5)	0.062 (3)	−0.020 (3)	0.007 (3)	−0.014 (4)
C16	0.071 (4)	0.100 (5)	0.086 (5)	−0.031 (4)	0.010 (3)	−0.024 (5)
C17	0.122 (8)	0.113 (7)	0.097 (6)	−0.055 (6)	0.026 (5)	−0.032 (5)
C18	0.093 (6)	0.193 (12)	0.076 (5)	−0.070 (7)	0.017 (5)	−0.032 (7)
C19	0.066 (5)	0.203 (11)	0.089 (7)	−0.037 (6)	−0.002 (4)	−0.040 (8)
C20	0.059 (3)	0.139 (7)	0.080 (5)	−0.018 (4)	−0.012 (3)	−0.003 (5)

### Geometric parameters (Å, °)

**Table:**

O1—C9	1.425 (7)	C10—H10A	0.9600
O1—H1O	0.8200	C10—H10B	0.9600
O2—C3	1.385 (7)	C10—H10C	0.9600
O2—H2O	0.8200	C11—C12	1.468 (10)
N1—C10	1.482 (7)	C11—H11A	0.9700
N1—C11	1.527 (7)	C11—H11B	0.9700
N1—C14	1.531 (7)	C12—C13	1.317 (3)
N1—C8	1.560 (6)	C12—H12	0.9300
C1—C6	1.387 (9)	C13—H13A	0.9300
C1—C2	1.396 (10)	C13—H13B	0.9300
C1—H1	0.9300	C13'—H13C	0.9300
C2—C3	1.377 (9)	C13'—H13D	0.9300
C2—H2	0.9300	C14—C15	1.495 (8)
C3—C4	1.372 (9)	C14—H14A	0.9700
C4—C5	1.373 (9)	C14—H14B	0.9700
C4—H4	0.9300	C15—C16	1.384 (11)
C5—C6	1.391 (9)	C15—C20	1.388 (10)
C5—H5	0.9300	C16—C17	1.429 (10)
C6—C7	1.487 (8)	C16—H16	0.9300
C7—C8	1.525 (8)	C17—C18	1.367 (17)
C7—H7A	0.9700	C17—H17	0.9300
C7—H7B	0.9700	C18—C19	1.365 (17)
C8—C9	1.520 (8)	C18—H18	0.9300
C8—H8	0.9800	C19—C20	1.361 (13)
C9—H9A	0.9700	C19—H19	0.9300
C9—H9B	0.9700	C20—H20	0.9300
			
C9—O1—H1O	109.5	N1—C10—H10A	109.5
C3—O2—H2O	109.5	N1—C10—H10B	109.5
C10—N1—C11	106.5 (4)	H10A—C10—H10B	109.5
C10—N1—C14	110.1 (5)	N1—C10—H10C	109.5
C11—N1—C14	109.2 (4)	H10A—C10—H10C	109.5
C10—N1—C8	112.9 (4)	H10B—C10—H10C	109.5
C11—N1—C8	110.0 (4)	C12—C11—N1	114.1 (5)
C14—N1—C8	108.1 (4)	C12—C11—H11A	108.7
C6—C1—C2	121.5 (7)	N1—C11—H11A	108.7
C6—C1—H1	119.2	C12—C11—H11B	108.7
C2—C1—H1	119.2	N1—C11—H11B	108.7
C3—C2—C1	119.1 (6)	H11A—C11—H11B	107.6
C3—C2—H2	120.5	C13—C12—C11	125.0 (9)
C1—C2—H2	120.5	C13—C12—H12	117.5
C4—C3—C2	120.4 (6)	C11—C12—H12	117.5
C4—C3—O2	123.0 (6)	C12—C13—H13A	120.0
C2—C3—O2	116.5 (6)	C12—C13—H13B	120.0
C3—C4—C5	119.8 (6)	H13A—C13—H13B	120.0
C3—C4—H4	120.1	H13C—C13'—H13D	120.0
C5—C4—H4	120.1	C15—C14—N1	116.3 (5)
C4—C5—C6	121.9 (6)	C15—C14—H14A	108.2
C4—C5—H5	119.0	N1—C14—H14A	108.2
C6—C5—H5	119.0	C15—C14—H14B	108.2
C1—C6—C5	117.1 (6)	N1—C14—H14B	108.2
C1—C6—C7	120.8 (6)	H14A—C14—H14B	107.4
C5—C6—C7	122.1 (5)	C16—C15—C20	118.7 (7)
C6—C7—C8	111.6 (5)	C16—C15—C14	118.9 (6)
C6—C7—H7A	109.3	C20—C15—C14	122.2 (7)
C8—C7—H7A	109.3	C15—C16—C17	120.1 (8)
C6—C7—H7B	109.3	C15—C16—H16	120.0
C8—C7—H7B	109.3	C17—C16—H16	120.0
H7A—C7—H7B	108.0	C18—C17—C16	117.2 (10)
C9—C8—C7	110.1 (5)	C18—C17—H17	121.4
C9—C8—N1	113.5 (4)	C16—C17—H17	121.4
C7—C8—N1	112.8 (4)	C19—C18—C17	123.4 (9)
C9—C8—H8	106.7	C19—C18—H18	118.3
C7—C8—H8	106.7	C17—C18—H18	118.3
N1—C8—H8	106.7	C20—C19—C18	118.5 (10)
O1—C9—C8	113.6 (5)	C20—C19—H19	120.7
O1—C9—H9A	108.8	C18—C19—H19	120.7
C8—C9—H9A	108.8	C19—C20—C15	122.0 (10)
O1—C9—H9B	108.8	C19—C20—H20	119.0
C8—C9—H9B	108.8	C15—C20—H20	119.0
H9A—C9—H9B	107.7		
			
C6—C1—C2—C3	1.0 (12)	C7—C8—C9—O1	51.2 (6)
C1—C2—C3—C4	−2.6 (12)	N1—C8—C9—O1	−76.3 (6)
C1—C2—C3—O2	179.8 (7)	C10—N1—C11—C12	175.1 (6)
C2—C3—C4—C5	3.1 (12)	C14—N1—C11—C12	−66.1 (7)
O2—C3—C4—C5	−179.5 (6)	C8—N1—C11—C12	52.5 (7)
C3—C4—C5—C6	−2.0 (11)	N1—C11—C12—C13	−144.3 (12)
C2—C1—C6—C5	0.1 (11)	C10—N1—C14—C15	61.4 (6)
C2—C1—C6—C7	179.2 (6)	C11—N1—C14—C15	−55.1 (7)
C4—C5—C6—C1	0.4 (10)	C8—N1—C14—C15	−174.8 (5)
C4—C5—C6—C7	−178.7 (6)	N1—C14—C15—C16	−93.4 (8)
C1—C6—C7—C8	−98.8 (8)	N1—C14—C15—C20	91.5 (9)
C5—C6—C7—C8	80.4 (8)	C20—C15—C16—C17	−2.0 (12)
C6—C7—C8—C9	65.0 (6)	C14—C15—C16—C17	−177.3 (7)
C6—C7—C8—N1	−167.1 (5)	C15—C16—C17—C18	1.0 (13)
C10—N1—C8—C9	67.4 (6)	C16—C17—C18—C19	0.4 (17)
C11—N1—C8—C9	−173.9 (4)	C17—C18—C19—C20	−0.6 (18)
C14—N1—C8—C9	−54.6 (6)	C18—C19—C20—C15	−0.5 (16)
C10—N1—C8—C7	−58.7 (6)	C16—C15—C20—C19	1.7 (14)
C11—N1—C8—C7	60.0 (6)	C14—C15—C20—C19	176.9 (8)
C14—N1—C8—C7	179.3 (5)		

### Hydrogen-bond geometry (Å, °)

**Table:**

*D*—H···*A*	*D*—H	H···*A*	*D*···*A*	*D*—H···*A*
O1—H1O···Br1	0.82	2.43	3.231 (4)	167
O2—H2O···Br1^i^	0.82	2.38	3.192 (5)	171

Symmetry codes: (i) *x*, *y*+1, *z*.
